# Use of e-Mental Health Tools for Suicide Prevention in Clinical Practice by Mental Health Professionals in NSW, Australia: Cross-Sectional Survey

**DOI:** 10.2196/64746

**Published:** 2025-03-26

**Authors:** Carol Hood, Sally Hunt, Alexandra P Metse, Rebecca K Hodder, Kim Colyvas, Rachel Sheather-Reid, David Duerden, Jenny Bowman

**Affiliations:** 1 School of Psychological Sciences The University of Newcastle Callaghan Australia; 2 Hunter Medical Research Institute Newcastle Australia; 3 Central Coast Mental Health Service Central Coast Local Health District Gosford Australia; 4 School of Health University of the Sunshine Coast Sippy Downs Australia; 5 Hunter New England Population Health Hunter New England Local Health District Wallsend Australia; 6 School of Medicine and Public Health The University of Newcastle Callaghan Australia

**Keywords:** suicide prevention, digital mental health, mental health professionals, peer support, internet, mobile apps, clinical practice, cross-sectional survey, Australia, e-mental health tools

## Abstract

**Background:**

Suicide is a significant global health concern. In the context of increased demand for mental health services and workforce shortages, exacerbated by the COVID-19 pandemic, electronic mental health (eMH) tools represent a promising means of augmenting mental health care generally and for suicide prevention specifically. A significant research gap exists however with respect to the use and uptake of eMH tools, especially electronic mental health tools for suicide prevention (eMH-SP).

**Objective:**

This study aimed to investigate the use of eMH tools by Australian mental health professionals, both in general and with respect to suicide prevention specifically, examining changes in use since COVID-19. Further, it explored factors associated with frequent use of eMH-SP, including sociodemographic and professional characteristics.

**Methods:**

A web-based cross-sectional survey was conducted across 15 local health districts (LHDs) in New South Wales, Australia, from May 2022 to July 2023. The sample was drawn from over 10,000 mental health professionals working in government services statewide. The survey explored the use of electronic mental health tools for general mental health issues (eMH-gen) and eMH-SP, explored the changes in the use of both since COVID-19, and used multivariable logistic regression to identify factors associated with the current use of eMH-SP.

**Results:**

Among 469 participants, increased use since COVID-19 was reported by over half (247/469, 52.7%) for eMH-gen, and by approximately one-third (141/386, 36.6%) for eMH-SP. The proportion reporting frequent use increased significantly from before to after COVID-19 for both eMH-gen (243/469, 51.8% to 283/469, 60.3%; *P<*.001) and eMH-SP (152/386, 39.4% to 170/385, 44.2%; *P=*.01). Since COVID-19, the most frequently used types of eMH tools for eMH-gen and eMH-SP, respectively, were information sites (231/469, 49.3% and 130/385, 33.8%), phone/online counseling (173/469, 36.9% and 130/385, 33.8%), and apps (145/469, 30.9% and 107/385, 27.8%). Professionals more likely to use eMH-SP frequently were females (odds ratio [OR] 3.32, 95% CI 1.88-5.87; *P*<.001) compared with males; peer workers (OR 2.17, 95% CI 1.0-4.71; *P*<.001) compared with nurses; those located in regional/rural LHDs (OR 1.65, 95% CI 1.04-2.61; *P*=.03) compared with metropolitan LHDs; and those practicing in emergency health care settings (OR 8.31, 95% CI 2.17-31.75; *P*=.03) compared with inpatient settings.

**Conclusions:**

The study’s findings highlight the increasing adoption of eMH tools and delivery of remote care by mental health professionals and provide valuable new insights into sociodemographic factors associated with the use of eMH for suicide prevention specifically. Continued research on the role eMH is playing is essential for guiding policy, optimizing resources, and enhancing mental health care and suicide prevention efforts.

## Introduction

### Background

Suicide is a leading global cause of death. In 2019, over 700,000 deaths were attributed to suicide worldwide; approximately one in every 100 deaths (1.3%) [1]. Suicide has a profound impact that extends beyond individuals who die by suicide, with those bereaved by suicide at higher risk of suicide themselves [2], and extensive social and economic impacts across the broader community. Suicide prevention initiatives are therefore crucial for decreasing suicide mortality and its wider impacts. The World Health Organization considers suicide preventable if effective evidence-based interventions, treatment, and support are provided in a timely manner [3].

In many nations including Australia, the mental health sector faces challenges in meeting a growing demand for services coupled with existing workforce shortages [4,5]. Pressure on the mental health sector has been further exacerbated by the COVID-19 pandemic (hereafter referred to as “COVID”), where effects have included: increased rates of depression, anxiety, and suicidal behavior [4,6]; reduced opportunity for in-person service contacts; and compounding of a chronic shortage of mental health professionals [7]. Mental health services need accessible and effective solutions that can be scaled up to reach larger numbers of people during times of increased demand.

The use of electronic mental health (eMH) tools to provide remote access to mental health services has emerged as an important part of the solution to bridge this service gap. eMH tools consist of digital technologies, including mobile apps, information sites, online peer support, web-delivered programs, and phone/online counseling [8], which are delivered remotely via electronic communication channels (eg, internet, telephone), and may be clinician-guided or self-managed to provide information or therapy [9]. eMH tools may be designed for standalone use or for integration into routine care to complement in-person support [10]. Their advantages have been noted to include expanded access to mental health services for difficult-to-reach populations, such as clients in geographically remote communities [11], those experiencing stigma or reluctance to engage in-person, or those who prefer anonymity [12]; reduced costs of mental health support [13]; and enhanced continuity or intensity of support (such as when blended with face-to-face care) [14].

Increasing evidence supports the effectiveness of eMH tools in reducing symptoms of various mental health conditions and in preventing suicide. For example, systematic reviews investigating internet-delivered cognitive behavior therapy have found it to be as effective as traditional face-to-face cognitive behavior therapy for anxiety and depressive disorders [15]; and to significantly reduce suicidal ideation compared with controls [16]. Although maintaining end user engagement can be challenging [17], and related to factors such as age, sex, and geographic location [18], end users generally report eMH tools to be acceptable, and have a positive attitude about their use [15,19].

Research reporting the prevalence of use and uptake of eMH tools has focused largely on “consumers” (people with lived experience of mental ill-health) as end users, using a range of measurement approaches and indicating widely varying levels of use [20]. A systematic review into the use of self-help apps and web-based eMH tools for depression, anxiety, or mood enhancement for example (n=11 studies, mostly from Australia and North America), found up to 88% of users had engaged at least minimally with an eMH tool; and that some tools achieved engagement of over 40,000 registrations or downloads monthly [21].

Fewer studies have investigated the prevalence of use and uptake of eMH tools by clinicians and peer workers (hereafter collectively referred to as “professionals”) in their work with consumers, with no systematic reviews synthesizing the findings of this limited body of literature being located to date. Two of the larger, more recent studies include a survey of 209 psychiatrists from 19 countries (including Australia) conducted prior to COVID, which indicated that 60% of psychiatrists did not recommend such tools to their patients [22]; and a repeated cross-sectional survey among a mix of psychologists, nurses, social workers, physicians and other professionals (n=1039) in the Netherlands, which found a significant increase in frequent use (monthly or weekly) of videoconferencing to deliver care since COVID [23].

While previous studies have explored the use of eMH tools for various mental health conditions, a crucial gap exists in understanding their use in suicide prevention. Prior to this research, we were able to identify only a recent, qualitative study of 15 Irish professionals describing the adoption of mobile apps for suicide prevention, which reported that while a majority (n=13) recommended apps to clients, only 3 professionals used the apps regularly in practice [24]. To evaluate the impact of eMH tools in the context of suicide prevention, it is vital to understand their use and uptake rates by professionals. This comprehensive study provides the first large-scale look at how mental health professionals use eMH tools for suicide prevention, offering new insights into current practices in this critical area of mental health care provision.

### Objectives

This study aimed to first investigate the use of eMH tools by mental health professionals employed in government services in Australia via a web-based survey, including electronic mental health tools for general mental health issues (eMH-gen) and electronic mental health tools for suicide prevention specifically (eMH-SP), and explicitly examine changes in use since COVID. Second, the study explored factors that may influence the frequency of eMH-SP use in practice, including sociodemographic and professional characteristics (age, sex, professional role, rurality of service location, work setting, primary mode of service delivery [whether predominantly in-person, remote, or a blend of both]).

## Methods

### Design and Setting

A web-based cross-sectional survey of mental health professionals working in the 15 local health districts (LHDs) of the public health system in New South Wales (NSW), Australia, was undertaken. LHD mental health services provide state-wide care for both acute and chronic mental health needs as follows: 6 metropolitan LHDs service the broader Sydney area, with the largest servicing over 1 million residents, while the smallest of 9 regional and rural LHDs services just over 30,000 residents (across nearly 200,000 sq km).

### Participants and Recruitment

Eligible participants were professionals (ie, nurses, occupational therapists, peer workers, psychologists, psychiatrists, and social workers) aged 18 years and older, currently providing mental health care within an NSW LHD service. Participants not conforming to these criteria were excluded from the study. Participants were invited to take part in the anonymous survey through emails sent from representatives within their organization. Participants were recruited between May 2022 and July 2023 for the survey, which was open for 8 weeks at each LHD. Three reminder emails were sent from most LHDs at fortnightly intervals to encourage participation (3 LHDs elected to send only 1 or 2 reminders).

Efforts to reduce bias included using neutral phrasing in survey questions, sending multiple reminders to encourage participation, and sampling across diverse demographics and work settings. No formal sample size and power calculation was carried out. Rather, an attempt was made to maximize the potential sample size by contacting all eligible professionals across the 15 NSW LHDs. The final sample size achieved was the result of the response rate to the survey.

### Survey Variables and Data Measurement

#### Overview

The web-based survey was developed through an iterative process that included reviewing existing literature, key stakeholder consultation, and review by an expert panel of mental health professionals (including an Aboriginal scholar and mental health clinician). The survey was deployed on the Research Electronic Data Capture (REDCap) platform [25], where it underwent piloting, with items revised for clarity and appropriateness. Survey data collection comprised predictor variables such as sociodemographic and professional descriptors; an exposure variable of the COVID period (before vs since); and key variables related to the primary outcomes regarding frequency of use of eMH-gen and eMH-SP.

#### Sociodemographic and Professional Characteristics

Demographic data collected included age, sex, and Aboriginal or Torres Strait Islander origin. Professional characteristics included professional role (nurse; occupational therapist; peer worker; psychiatrist; psychologist; social worker; other), length of service as a mental health professional, suicide prevention training attendance within the previous 3 years (yes/no), LHD of employment (multiple selections permitted from Central Coast; Far West; Hunter New England; Illawarra Shoalhaven; Mid North Coast; Murrumbidgee; Nepean Blue Mountains; Northern NSW; Northern Sydney; South Eastern Sydney; South Western Sydney; Southern NSW; Sydney; Western NSW; Western Sydney), work setting (inpatient; emergency; community; other), and area of mental health practice (multiple selections permitted from: Aboriginal; adult; child and adolescent; consultation liaison; forensic; improvement or service development; neuropsychiatry or neuropsychology; older people; perinatal; special projects; other).

The primary mode of health care delivery was calculated based on participants’ ratings of the proportion of care (0% to 100%) they delivered via in-person and remote (phone; video; online text) modes. Responses were sought with respect to delivery mode prior to COVID (ie, prior to the World Health Organization declaration of the pandemic on 11 March 2020 [26]) as well as currently (ie, at the time of survey completion).

#### Use of e-Mental Health Tools

The survey defined eMH tools as “digital resources and tools delivered via online or telephone technologies that support provision of mental health care”, and categorized the types of eMH tools according to the e-Mental Health in Practice website [8], a comprehensive directory of evidence-based Australian, digital mental health resources. The categories of tool types include apps, information sites, online peer support, online self-directed programs, online coach-assisted programs, and phone/online counseling. The e-Mental Health in Practice directory groups eMH tools by topic, including general issues such as mental health conditions, relationship issues, or trauma (eMH-gen); and suicide prevention (eMH-SP).

Participants were asked about their use of eMH tools in terms of (1) change in the extent of eMH-gen and eMH-SP use overall since COVID, rated on a 5-point Likert scale (reduced considerably; reduced somewhat; no change; increased somewhat; increased considerably); (2) frequency of use, based on participants’ most frequently used type of eMH tool, rated on a 5-point Likert frequency scale (never, less than monthly, monthly, weekly, daily; the responses were sought separately for eMH-gen and eMH-SP, and use was compared before and since COVID for both); and (3) types of eMH used, based on the frequency of use for each individual tool type rated on the same 5-point Likert scale (never to daily), with responses sought separately for the types of eMH-gen and eMH-SP, and use compared before and since COVID for both.

### Statistical Methods

Descriptive statistics were used to characterize the sample and explore the prevalence of the use of eMH, both eMH-gen, and eMH-SP. Continuous data were presented as means and SDs, and categorical data as frequencies and corresponding percentages. Condensed response categories were created based on age, length of service, LHD location, and delivery mode.

Binary categories were created for the frequency of eMH use and for types of eMH tools (rarely [never, less than monthly, and monthly], frequently [weekly and daily]). McNemar tests were used to assess the significance of differences before and since COVID. To assess differences in delivery modes before and since COVID, paired data were first assessed for normality using Shapiro-Wilk tests and skewness values. Based on these assessments, Wilcoxon signed rank tests (for non-normally distributed data) were carried out, with the α level set to .01 after applying the Bonferroni correction to adjust for multiple comparisons, reducing the risk of type I errors. To address the potential impact of participants dropping out early before completing questions on eMH-SP, such participants were compared with survey completers using chi-square analyses to examine for differences in sociodemographic characteristics (age, sex, and Aboriginal or Torres Strait Islander origin) and professional attributes (professional role, length of service, suicide prevention training, LHD location, and work setting).

Univariate chi-square tests were conducted to identify associations between categorical variables, in this case, factors suggested by the literature as theoretically likely to be associated with frequent eMH use. The following independent variables were considered: age, sex, professional role, LHD location, work setting, and delivery mode. Univariate analysis was carried out on a condensed 3-point Likert scale (never, occasionally [monthly or less than monthly], and frequently [weekly or daily]) using chi-square analysis to test the significance of the independent variables. Those that were significant were entered into a multiple-variable binary logistic regression model. To enable this, the outcome variable was dichotomized into binary categories (rarely [never or occasionally] and frequently) for the multivariable analysis. The independent variables considered in the logistic regression model were all the variables examined in the univariate analysis, as they were all significant. None of the variables were removed from the model to comprehensively assess the combined effects of these variables. The study was conducted and reported in accordance with the STROBE (Strengthening the Reporting of Observational Studies in Epidemiology) guidelines [27]. Data were analyzed using the IBM Statistical Package for the Social Sciences (version 29; IBM Corp).

### Ethics Approval

Ethics approval was received from the Hunter New England Human Research Ethics Committee (approval 2021/ETH00613) and the University of Newcastle Human Research Ethics Committee (approval H-2022-0034). Local research governance authorization was obtained from each participating LHD. Participants received an email invitation with an information statement outlining the study’s objectives, procedures, and potential risks. The information statement was also on the survey website, which participants reviewed before consenting to participate. Completing the web-based survey questions implied informed consent. Participation was voluntary, with the option to opt out at any time. Responses were anonymous, and the data were stored in encrypted electronic databases accessible only to the research team. No financial compensation was provided.

## Results

### Participants

#### Overview

**Figure 1:**
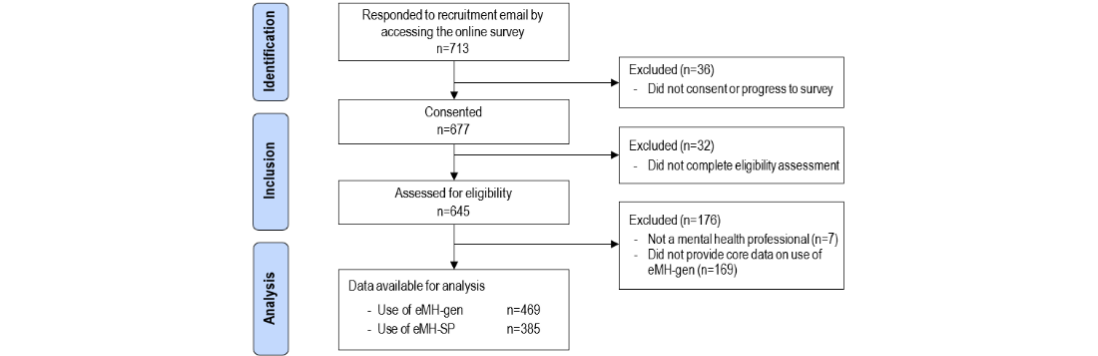
Flowchart of the study population. eMH: electronic mental health; eMH-gen: electronic mental health tools for general mental health issues; eMH-SP: electronic mental health tools for suicide prevention.

Participants were drawn from all 15 LHDs across NSW, with over 10,000 mental health staff invited to participate (ranging from 33 to 1400 per LHD). Of the 713 respondents who accessed the survey, 677 consented, of whom 645 provided information allowing assessment of eligibility (with 7 then excluded). A final sample of 469 participants provided data on the use of eMH-gen; with 385 participants providing data that enabled inclusion in analysis for the use of eMH-SP (Figure 1). The sociodemographic and professional characteristics of the 469 participants whose data were analyzed in this study are reported in Table 1. No significant differences were found in sociodemographic or professional characteristics between participants who dropped out early and those who completed all survey questions.

**Table 1:** Participant sociodemographic and professional characteristics (N=469).

Individual sociodemographic and professional characteristics		Values
Age (years), mean (SD)		46.2 (12.7)
Age (years), range		22-77
**Age (years), n (%)**		
	18-34	103 (22)
	35-54	225 (48)
	55 and older	141 (30.1)
**Sex** **, n (%)**		
	Female	339 (72.3)
	Male	127 (27.1)
	Prefer not to disclose	3 (0.6)
**Aboriginal or Torres Strait Islander origin** **, n (%)**		
	Not Aboriginal or Torres Strait Islander	444 (94.7)
	Aboriginal	13 (2.8)
	Aboriginal or Torres Strait Islander	1 (0.2)
	Do not know	3 (0.6)
	Prefer not to disclose	8 (1.7)
**Professional role** **, n (%)**		
	Nurse	216 (46.1)
	Psychologist	55 (11.7)
	Social worker	53 (11.3)
	Peer worker	47 (10)
	Occupational therapist	37 (7.9)
	Psychiatrist	30 (6.4)
	Other (eg, welfare officer, mental health practitioner)	31 (6.6)
Length of service (years), mean (SD)		14.3 (11.6)
Length of service (years), range		1-53
**Length of service, n (%)**		
	0-5 years	133 (28.4)
	6-10 years	92 (19.6)
	11-20 years	131 (27.9)
	21 years and over	113 (24.1)
Suicide prevention training within last 3 years, n (%)		294 (62.7)
**Location of mental health service^a^, n (%)**		
	Regional or rural LHD^b^	260 (55.4)
	Metropolitan LHD	204 (43.5)
	LHD not disclosed	5 (1.1)
**Work setting, n (%)**		
	Community	277 (59.1)
	Inpatient	139 (29.6)
	Emergency	18 (3.8)
	Other (eg, a combination of any or all the above)	35 (7.5)
**Area of practice (multiple selections permitted), n (%)**		
	Adult	340 (72.5)
	Child and adolescent	138 (29.4)
	Older people	113 (24.1)
	Aboriginal	99 (21.1)
	Forensic	46 (9.8)
	Perinatal	38 (8.1)
	Consultation liaison	37 (7.9)
	Special projects	33 (7)
	Improvement or service development	24 (5.1)
	Neuropsychiatry or neuropsychology	8 (1.7)
	Other	31 (6.6)

^a^One participant worked at both a metropolitan and a regional/rural LHD (assigned to the metropolitan LHD), and several participants worked across multiple (2-3) metropolitan or regional/rural LHDs).

^b^LHD: local health district.

#### Primary Mode of Health Care Delivery

Initial analyses revealed significant deviations from normality in the data (Shapiro-Wilk tests: all *P*<.001; skewness values >±1, except one variable with skewness of –0.569), necessitating the use of Wilcoxon signed rank tests. The Wilcoxon signed rank tests revealed significant changes in the proportion of care delivered via the different modes (in-person and remote) from pre-COVID to since COVID. In-person care remained the primary mode of delivery but decreased significantly from 78.6% pre-COVID to 66.5% since COVID (*z*=11.07, *P<*.001, N=467). Conversely, all modes of remote care delivery increased, with significant rises in phone-based care from 13.2% to 17.0% (*z*=6.96, *P<*.001, N=463), video-based care from 2.7% to 10.3% (*z*=10.98, *P<*.001, N=458), and online text-based care from 2.3% to 3.7% (*z*=2.71, *P=*.007, N=462). While most participants used a combination of 2 to 3 delivery modes, the percentage using exclusively in-person delivery modes decreased significantly from 33.9% pre-COVID to 24.5% since COVID (*P<*.001).

### Use of e-Mental Health Tools

#### Overview

This section presents findings on mental health professionals’ use of eMH tools. The frequency of eMH use (including both detailed frequency levels and broader binary categories of frequently and rarely used), and the types of eMH tools they frequently use are presented in Table 2.

**Table 2:** Frequency of use of e-Mental Health (eMH) tools and types of eMH used frequently (daily or weekly), for electronic mental health tools for general mental health issues (eMH-gen) and electronic mental health tools for suicide prevention specifically (eMH-SP), measured both before and since the onset of COVID.

					Pre-COVID, n (%)		Since COVID, n (%)		Significance (McNemar test *P* value)
**Frequency of use of eMH-gen**									
	Total, n				469		469		—^a^
	Never				28 (6)		29 (6.2)		—
	Less than monthly				80 (17.1)		50 (10.7)		—
	Monthly				118 (25.2)		107 (22.8)		—
	Weekly				164 (35)		177 (37.7)		—
	Daily				79 (16.8)		106 (22.6)		—
	**Combined categories of use**								
		Frequently (weekly or daily)	243 (51.8)			283 (60.3)		<.001	
		Rarely (never or <monthly or monthly)	226 (48.2)			186 (39.7)		—	
**Frequency of use of eMH-SP**									
	Total, n				386		385		—
	Never				53 (13.7)		45 (11.7)		—
	Less than monthly				91 (23.6)		80 (20.8)		—
	Monthly				90 (23.3)		90 (23.4)		—
	Weekly				95 (24.6)		106 (27.5)		—
	Daily				57 (14.8)		64 (16.6)		—
	**Combined categories of use**								
		Frequently (weekly or daily)		152 (39.4)			170 (44.2)		.01
		Rarely (never or <monthly or monthly)		234 (60.6)			215 (55.8)		—
**Types of eMH-gen used frequently** **(daily or weekly)**									
	Total, n				469		469		—
	Information sites				180 (38.4)		231 (49.3)		<.001
	Phone/online counseling				168 (35.8)		173 (36.9)		.67
	Apps				120 (25.6)		145 (30.9)		.01
	Online self-directed programs				65 (13.9)		90 (19.2)		<.001
	Online peer support				47 (10)		68 (14.5)		.002
	Online coach-assisted programs				43 (9.2)		60 (12.8)		.01
	Other				37 (7.9)		50 (10.7)		.04
**Types of eMH-SP used frequently** **(daily or weekly)**									
	Total, n				386		385		—
	Information sites				114 (29.5)		130 (33.8)		.03
	Phone/online counseling				113 (29.3)		130 (33.8)		.01
	Apps				57 (14.8)		107 (27.8)		<.001
	Online self-directed programs				42 (10.9)		62 (16.1)		.002
	Online peer support				43 (11.1)		74 (19.2)		<.001
	Online coach-assisted programs				26 (6.7)		43 (11.2)		<.001
	Other				19 (4.9)		33 (8.6)		<.001

^a^Not applicable.

#### Changes in Use of eMH

Over half the participants (247/469, 52.7%) reported an increase in their use of eMH-gen since COVID; 43.3% (n=203) reported no change, and a small minority (19/469, 4%) reported a decrease in use. Regarding eMH-SP, over one-third of participants (141/385, 36.6%) reported an increase in their use, over half reported no change in their frequency of use (226/385, 58.7%), while less than 5% (n=18) reported a reduction in the frequency of eMH-SP use.

#### Frequency of Use of eMH

Among participants, 60.3% (n=283) reported currently using eMH-gen frequently, compared with 51.8% (n=243) pre-COVID, a significant increase (*P<*.001). Similarly, 44.2% (n=170) reported frequently using eMH-SP compared with 39.4% (n=152) pre-COVID, a significant increase (*P=*.01). Comparing frequent use of eMH-gen with eMH-SP since COVID, the difference was also significant (*P<*.001).

#### Types of eMH Tools Used

Of the 6 types of eMH tools assessed, the 3 eMH-gen currently used most frequently were information sites (231/469, 49.3%), phone/online counseling (173/469, 36.9%), and apps (145/469, 30.9%), with significant increases since COVID in both information sites (*P<*.001) and apps (*P=*.01). Among these 6 types of tools, the 3 eMH-SP currently used most frequently, all of which showed significant increases since COVID, were information sites (130/385, 33.8%; *P=*.03), phone/online counseling (130/385, 33.8%; *P=*.01), and apps (107/385, 27.8%; *P<*.001).

#### Sociodemographic and Professional Characteristics Associated With the Use of eMH-SP in Current Practice

Current frequency of use of eMH-SP was classified into one of 3 outcome categories (never, occasionally, and frequently), with possible associations explored with sociodemographic and professional characteristics (Table 3). Chi-square test results indicate significant univariate associations between all of the factors and the frequency of use of eMH-SP. Professionals of younger age, female sex, professional role of peer worker or social worker, working in regional or rural LHDs, in emergency health care settings, and using at least 1 remote delivery mode (as opposed to in-person mode only) were more likely to report current frequent (weekly or daily) use of eMH-SP.

Multivariable binary logistic regression was then used (categories: rarely; frequently) to further explore the relationship between these factors and frequent use of eMH-SP (ie, on a daily or weekly basis), as presented in Table 4. The overall model was found to be significant (χ^2^_14_=70.89, n=378, and *P<*.001). Age (*P=*.06) and delivery mode (*P=*.17) were no longer significant, while significance was retained for all other factors. Frequent use of eMH-SP was 3.32 times more likely for females than males, 2.17 times more likely for peer workers than nurses, 1.65 times more likely for regional/rural LHDs than metropolitan LHDs, and 8.31 times more likely for emergency settings than inpatient settings.

**Table 3:** Sociodemographic and professional characteristics associated with the frequency of use of electronic mental health tools for suicide prevention (eMH-SP) in current practice (N=385, percentages by rows).

		Never, n (%)	Occasionally (monthly or less), n (%)	Frequently (weekly or daily), n (%)	Chi-square (*df*)		*P* value	
**Age (years)**						13.65 (4)		.008
	18-34 (n=79)	7 (8.9)	34 (43)	38 (48.1)				
	35-54 (n=185)	15 (8.1)	78 (42.2)	92 (49.7)				
	55 and older (n=121)	23 (19)	58 (47.9)	40 (33.1)				
**Sex**						18.76 (4)		*<*.001
	Female (n=278)	28 (10.1)	109 (39.2)	141 (50.7)				
	Male (n=104)	16 (15.4)	60 (57.7)	28 (26.9)				
	Prefer not to disclose (n=3)	1 (33.3)	1 (33.3)	1 (33.3)				
**Professional role**						34.72 (12)		*<*.001
	Peer worker (n=40)	0 (0)	14 (34.1)	26 (65.9)				
	Social worker (n=40)	6 (15)	11 (27.5)	23 (57.5)				
	Nurse (n=179)	24 (13.4)	81 (45.3)	74 (41.3)				
	Psychologist (n=47)	2 (4.3)	27 (57.4)	18 (38.3)				
	Psychiatrist (n=24)	4 (16.7)	14 (58.3)	6 (25)				
	Occupational therapist (n=28)	7 (25.9)	15 (55.6)	6 (18.5)				
	Other (n=27)	2 (7.4)	8 (29.6)	17 (63)				
**LHD^a^ location (n=381)**						10.41 (2)		.005
	Regional/rural LHD (n=213)	21 (9.9)	82 (38.5)	110 (51.6)				
	Metropolitan LHD (n=168)	23 (13.7)	86 (51.2)	59 (35.1)				
**Work setting**						14.24 (6)		.03
	Emergency (n=14)	1 (7.1)	3 (21.4)	10 (71.4)				
	Community (n=226)	20 (8.8)	98 (43.4)	108 (47.8)				
	Inpatient (n=113)	20 (17.7)	56 (49.6)	37 (32.7)				
	Other (>1 setting, n=32)	4 (12.5)	13 (40.6)	15 (46.9)				
**Delivery mode**						18.21 (2)		*<*.001
	In-person or remote (n=295)	24 (8.1)	129 (43.7)	142 (48.1)				
	In-person only (n=90)	21 (23.3)	41 (45.6)	28 (31.1)				

^a^LHD: local health district.

**Table 4:** Binary logistic regression model results with regression coefficients (B), standard errors (SE), odds ratios (OR), and 95% CIs for factors associated with the likelihood of frequent use of electronic mental health tools for suicide prevention (eMH-SP) in current practice (N=378).

		Values, n (%)	B	SE	*P* value	Odds ratio (95% CI)	Likelihood ratio test			
							Chi-square (*df*)		*P* value	
**Age group (years)**								5.570 (2)		.06
	18-34	78 (20.6)	0.70	0.35	.04	2.01 (1.02-3.97)				
	35-54	183 (48.4)	0.56	0.27	.04	1.75 (1.03-2.99)				
	55 and older (reference)	117 (31)	0	—^a^	—	1.00^b^				
**Sex**								18.791 (1)		<.001
	Female	276 (73)	1.20	0.29	<.001	3.32 (1.88-5.87)				
	Male (reference)	102 (27)	0	—	—	1.00^b^				
**Professional role**								22.525 (6)		<.001
	Peer worker	40 (10.6)	0.77	0.40	.05	2.17 (1.0-4.71)				
	Social worker	39 (10.3)	0.16	0.40	.68	1.18 (0.54-2.56)				
	Psychologist	47 (12.4)	–0.50	0.37	.18	0.61 (0.29-1.27)				
	Psychiatrist	22 (5.8)	–0.88	0.58	.13	0.42 (0.13-1.30)				
	Occupational therapist	27 (7.1)	–1.41	0.53	.01	0.24 (0.09-0.69)				
	Other	26 (6.9)	0.70	0.47	.14	2.02 (0.80-5.08)				
	Nurse (reference)	177 (46.8)	0	—	—	1.00^b^				
**LHD^c^ location**							4.561 (1)		.03	
	Rural/regional	211 (55.8)	0.50	0.23	.03	1.65 (1.04-2.61)				
	Metropolitan (reference)	167 (44.2)	0	—	—	1.00^b^				
**Setting**							9.294 (3)		.03	
	Emergency	14 (3.7)	2.12	0.68	.002	8.31 (2.17-31.75)				
	Community	222 (58.7)	0.67	0.27	.01	1.95 (1.15-3.32)				
	Other (eg, >1 setting)	31 (8.2)	0.65	0.46	.17	1.91 (0.77-4.73)				
	Inpatient (reference)	111 (29.4)	0	—	—	1.00^b^				
**Delivery mode**							1.851 (1)		.17	
	In-person or remote	288 (76.2)	0.51	0.38	.18	1.66 (0.80-3.47)				
	In-person only (reference)	90 (23.8)	0	—	—	1.00^b^				

^a^Not applicable.

^b^The parameter of B for each of the reference categories is set to 0 because it is redundant and there are no confidence intervals for the reference categories.

^c^LHD: local health district.

## Discussion

### Principal Findings

This study invited mental health professionals in government mental health services across NSW Australia to take part in a survey with respect to the use of eMH tools in their professional practice. It focused particularly on the use of eMH-SP, given the urgent need for strengthening suicide prevention efforts in the face of consistently high suicide rates. Understanding the uptake of eMH is especially important in the context of challenges in meeting increasing demand for mental health care, both generally and for suicide prevention; with demand heightened further by the COVID pandemic. Study findings reflected a shift toward the increasing incorporation of remote modes of care delivery (video, phone, online text) since COVID, with the proportion reporting delivering care solely “in-person” declining from one-third to one-quarter.

Over half (247/469, 52.7%) of participants reported an increase in their use of eMH-gen since COVID, while 43.3% (n=203) reported no change. Over one-third (141/386, 36.6%) similarly reported their use of eMH-SP had increased, with 58.7% (n=226) reporting their use remained unchanged. A majority (283/469, 60.3%) of professionals reported frequent use of eMH-gen (at least weekly); a proportion that had increased by approximately 10% since COVID. While a lesser proportion of participants reported frequent use of eMH-SP specifically, both before and since COVID, there was still a notable increase in its use. Nearly half (170/386, 44.2%) of the sample reported frequent use of eMH-SP since COVID, up from 39.4% (n=152) prior to the pandemic. Approximately half of the participants (231/469, 49.3%) reported information sites as the most frequently used type of eMH-gen, followed by phone/online counseling (173/469, 36.9%). The same types of tools were also most frequently used with respect to suicide prevention, with approximately one-third reporting information sites and phone/online counseling as their most frequently used tools. Since COVID, the use of phone/online counseling for suicide prevention has increased significantly, in contrast to its use for general mental health issues. This shift may have been driven by the pandemic, which limited in-person counseling and support for suicide prevention, instead prompting increased use of eMH-SP. The most marked increases in the frequency of use by types of eMH tools were observed for information sites in eMH-gen (from 180/469, 38.4% to 231/469, 49.3%), and for suicide prevention apps in eMH-SP (from 57/385, 14.8% to 107/385, 27.8%). This increase in overall use is consistent with the rapidly increasing availability and promotion of digital mental health resources in practice [28].

A small but consistent proportion of professionals (≈6% both pre-COVID and currently) reported never using eMH-gen, while roughly double this proportion (≈14% pre-COVID and 12% currently) reported never using eMH-SP. Over half of respondents reported no or very limited (monthly or less) use of eMH-SP currently, as compared with approximately one-third who reported no or very limited use of eMH-gen. To some extent, this lesser use for suicide prevention specifically might be related to a lack of knowledge about what eMH-SP are available and are indicated for use with clients experiencing suicidal ideation [29], as well as concerns about the safety and effectiveness of these tools in managing suicidal crises [30]. It is also however likely explained in part by some clinicians encountering clients at high risk of suicide relatively infrequently in their clinical practice. This is lent support by the finding that the use of eMH-SP did differ by setting; frequent use being highly reported by the small number of survey respondents who worked in emergency settings (less than 4% of participants), where presentations involving suicidal ideation and suicide attempts would be more common.

Frequent use of eMH-SP was also more likely for female mental health professionals as compared with males. While this finding aligns with the general trend of higher digital tool use among females [31], no corroborating evidence specific to mental health professionals was found in the existing literature. The finding that some professional groups, most notably peer workers and social workers, were more likely to report frequent use of eMH-SP than others, might suggest that daily routines and demands of the various professional roles influence levels of use of these tools, but further research is needed to elucidate this relationship. Notably, all peer workers used eMH-SP to some extent, with almost two-thirds using these tools frequently. It might be speculated that the finding of higher use in regional or rural LHDs may be due to participants in such locations being more likely to promote eMH adoption to compensate for limited access to mental health resources compared with those in metropolitan LHDs [10].

The delivery of mental health care and suicide prevention is continuing to evolve, with the findings of this study supporting previous research suggesting that remote delivery modes and the use of eMH tools are becoming more common [32]. This raises questions about potential barriers to eMH adoption, especially in suicide prevention, where clinicians may face added safety and ethical concerns about recommending appropriate eMH tools to consumers at heightened risk of suicide [33]. It also creates the need for mental health services to respond and provide appropriate leadership, infrastructure access, and training to allow mental health professionals to optimize the use of eMH tools, despite the challenges. The rapid growth in the number of available eMH-SP, coupled with the variable expertise involved in their development, has resulted in inconsistent quality across these tools [34,35]. The use of eMH is already quite significant and likely to continue rising, and brings with it the need for clear, best practice guidelines to direct mental health professionals toward the most effective and safest eMH-SP for clinical practice.

This study offers valuable insights into eMH use by diverse mental health professionals in NSW, both before and since the COVID pandemic. One significant strength is its comprehensive reach, encompassing all 15 LHDs across NSW and inviting over 10,000 mental health staff to participate. However, there are limitations to consider for the study. The results are based on a self-report survey, which may exhibit response bias. The research was conducted in a single Australian state, limiting the generalizability of the results to other contexts. However, NSW is the most populous state with the highest number of mental health professionals [36]. The survey had a modest participation rate (4.7%), which nevertheless equated to a substantial sample of 469 participants. The dropout rate was 17.9%, with 84 of the 469 eligible participants failing to complete the survey, although there were no significant differences in sociodemographic or professional characteristics between survey dropouts and completers. Self-selection bias may have been introduced, with those who opted to complete the survey likely to have a stronger interest in eMH and be more likely to use it.

Despite such limitations, this study makes a significant contribution to the field by systematically documenting real-life practices of mental health professionals’ use of eMH tools since the COVID pandemic, with a focus on suicide prevention. Unlike previous research, this study provides a comprehensive, quantitative overview that not only assesses mental health professionals’ adoption of eMH tools but also provides important insights about sociodemographic factors that may influence the uptake of these tools into practice. Building on previous qualitative insights, this study provides a more comprehensive, data-driven understanding of eMH tool use in suicide prevention. Using a quantitative approach, the study offers statistically robust evidence beyond the limited scope of existing literature.

### Conclusion

It is vitally important to continue to explore avenues for increasing timely access to quality mental health support, particularly in the area of suicide prevention. In order to promote appropriate uptake of eMH-SP in practice, it is imperative to understand the perceived benefits, challenges, and barriers to use. Future research could explore the factors that clinicians report influence the adoption of eMH-SP in greater detail, possibly using quantitative or qualitative approaches with selected mental health professionals and perhaps different professional groups, to gain deeper insights into their experiences and motivations for using or not using eMH tools for suicide prevention. Implementation research is needed to identify strategies for mental health services to support clinicians in their optimal use of these tools, which may include, for instance, best practice guidelines. Research in these areas is essential for guiding policy and resource allocation, improving the quality of mental health care, and enhancing suicide prevention efforts in this critical area of public health.

## Abbreviations

eMH: electronic mental health
eMH-gen: electronic mental health tools for general mental health issues
eMH-SP: electronic mental health tools for suicide prevention
LHD: local health district
NSW: New South Wales
OR: odds ratio
REDCap: Research Electronic Data Capture
STROBE: Strengthening the Reporting of Observational Studies in Epidemiology

## Data Availability

The datasets generated or analyzed during this study are not publicly available due to ethical restrictions and privacy considerations (eg, data containing potentially identifying information), but are available from the corresponding author on reasonable request.
